# *mec-A-*mediated Resistance in *Staphylococcus aureus* in a Referral Hospital, Tehran, Iran

**DOI:** 10.5812/jjm.9181

**Published:** 2014-04-01

**Authors:** Afsaneh Rajabiani, Fatemeh Kamrani, Mohammad Ali Boroumand, Hiva Saffar

**Affiliations:** 1 Department of Pathology, Shariati Hospital, Tehran University of Medical Sciences, Tehran, IR Iran; 2 Department of Pathology, Tehran Heart Center, Tehran University of Medical Sciences, Tehran, IR Iran

**Keywords:** Community-Acquired Infections, Meticillin, Polymerase Chain Reaction, *Staphylococcus aureus*

## Abstract

**Background:**

The emerge of rapid and accurate detection of Meticillin-Resistant *Staphylococcus aureus *(MRSA) has been highlighted.

**Objectives:**

The current study evaluated the prevalence of *mec-A *gene in biological specimens of various medical wards, in order to determine any possible relationship.

**Patients and Methods:**

Using traditional culture methods, 250 isolates were detected. The prevalence of *mec-A* mediated resistance was evaluated by PCR method.

**Results:**

Among 98 isolates (39.2%) with resistant inhibition zones, 92 isolates carried *mec-A *gene and were considered as MRSA. Significantly higher rate of MRSA was observed in the specimens from emergency department and intensive care unit (P value < 0.001). Although, the prevalence of MRSA was higher in patients with history of previous hospital admission within the past three months (P = 0.006), but only one case with the same history was hospitalized in the emergency ward that was among the wards with the highest rate of MRSA.

**Conclusions:**

The study findings show that, although there is higher rate of MRSA infection in patients with history of hospitalization, but even in cases without any history of medical admission, more detailed questions emphasizing on receiving any recent health care should be asked in a referral hospital, in order to determine the true community-acquired MRSA.

## 1. Background

The first case of meticillin-resistant *Staphylococcus aureus *(MRSA), was reported in the early 1960, which has become an increasing problem in health care centers and communities (1). The *mec-A* gene, is a genetic element found in MRSA strains. It encodes an additional penicillin binding protein PBP2a, which has a low affinity to all ß-lactam antibiotics and is responsible for the meticillin resistance of MRSA strains (2, 3). Since MRSA are significant pathogens which impose a burden on sub-acute and chronic care facilities (4), rapid and accurate detection of MRSA is of great importance in order to appropriately manage patients, hospitals and community epidemiology (5).

Detection of meticillin-resistance (MR) in the clinical laboratory settings is related to environmental conditions such as temperature, pH, incubation time or salt concentration of the media (6). Furthermore, conditional or heterogeneous expression of MR can cause ambiguity in routine susceptibility tests (6). These facts justify the need for a sensitive, rapid, and accurate method. Therefore, detection of *mec-A *gene by PCR method has been considered as the reference method (7).

## 2. Objectives

The current study evaluated the prevalence of MRSA infections and the *mec-A* gene in different biological specimens collected from patients in various medical wards, in order to determine any possible relation. The study was conducted in Shariati Hospital, which is a general and referral health center in Tehran.

## 3. Materials and Methods

In a one-year study (2011), a total of 250 isolates were detected by traditional culture methods used to recognize Staphylococcus strains with further confirmation by standard phenotypical methods including catalase, coagulase and DNase. Following confirmation of bacterial types, the bacteria were analyzed using disc diffusion method and 1 µg Oxacillin discs (Mast Discs^TM^), in order to assess the bacterial sensitivity to meticillin. The interpretation was made according to CLSI standards (8). Then, the presence of *mec-A* gene was determined by PCR assay.

### 3.1. PCR Assay

Boiling method for DNA extraction (9) with some modifications was applied. Briefly, single staphylococcal colony was selected from 24 hours Blood agar medium and washed twice with TE buffer (Tris 10 mM, EDTA 1 mM, pH 8.0). The final pellet was resuspended in 200 µL of sterile distilled water and boiled for 10 minutes in 100°C boiling water, then centrifuged for 3 minutes at 8000 g to precipitate the bacterial debris. Supernatant was transferred to a new DNase and RNase free sterile tube. Three µL of the supernatant was used as template for PCR reaction.

The *mec-A* gene was amplified using 5' GTA GAA ATG ACT GAA CGT CCG ATA A-3' as forward and 5'- CCA ATT CCA CAT TGT TTC GGT CTA A3' as reverse primers (10) (BioRad MJ MiniTM PCR system). The amplification condition for *mec-A* gene consisted of an initial denaturation step at 94°C for 4 minutes and 35 cycles of 94°C for 45 seconds, 50°C for 45 seconds and 72°C for 1 minute with final extension at 72°C for 2 minutes. Finally, PCR products (533 base pair) were run on a 1% agarose gel electrophoresis.

A tube containing all PCR reaction mixture and human DNA as template was used as negative control and a known case of MRSA isolate was used as positive control. The patient records were studied to collect the data regarding their gender, age, admission ward, history of prior admission and infection site. SPSS software (SPSS Incversion17, Illinois, USA) was used for data analysis.

## 4. Results

A total of 250 isolates of *S. aureus *were studied, 135 and 115 of those isolates were isolated from female and male patients, respectively. The mean age of patients was 38.7 ± 12.7 years. Among 98 isolates (39.2%) with resistant inhibition zones, 92 carried *mec-A *gene which were considered as MRSA. Thus, the prevalence of *mec-A* mediated resistance was estimated about 36.8% . 

The patients were classified with respect to the admission ward. For a better determination of any possible relationship between the prevalence of MRSA and admission ward, the medical wards were categorized into four main groups including Internal medical wards (Respiratory disease, Nephrology, Endocrinology, Gastroenterology, Bone Marrow Transplant and General internal medicine), Intensive Care Units, Surgical wards (General surgery, Orthopedics, Neurosurgery, Gynecology) and Emergency department. The prevalence of MRSA in each group is summarized in Table 1. A significantly higher rate of MRSA was observed in specimens from emergency department and intensive care unit (P < 0.001). Also, the prevalence of MRSA infection was found to be higher in patients with the history of hospital admission within the past three months (P = 0.006). Data are summarized in Table 2.

**Table 1.** The Prevalence of *mec-A* Gene Isolated From Patients Admitted to the Medical Wards ^a^

Prevalence of *mec-A *Gene				Total (n = 250)	P Value
Emergency Unit (n = 46)	Internal Medical Wards (n = 111)	Intensive Care Units (n = 33)	Surgery Units (n = 60)		
30 (65.2)	25 (22.5)	22 (66.7)	15 (25.0)	92 (36.8)	< 0.001

^a^ Data are presented as No. (%)

**Table 2.** The Prevalence of *mec-A *gene Isolated From Patients with the History of Hospital Admission^a^

Prevalence of *mec-A *Gene		Total (n=250)	P Value
Positive History for Previous Admission (n = 22)	Negative History for Previous Admission (n = 228)		
14 (63.6)	78 (34.2)	92 (36.8)	0.006

^a^ Data are presented as No. (%)

## 5. Discussion

*S. aureus* is considered as one of the most common bacteria in the clinical practice (6) which has shown an increasing incidence of antibiotic resistance over the past decade (3); if they are not diagnosed or managed early and properly enough, they can cause a range of life threatening conditions (3). The outcomes of clinical studies have shown that reducing the diagnosis time decreases the mortality and morbidity rates (3).

MRSA is distributed widely through different parts of the world. For example, surveillance results of blood stream infections showed noticeable variability range of 1 to 50% in different countries in European Union (11), and 20-40% and even up to 80% in some centers in India (6). In Saudi Arabia 42 0ut 0f 119isolates of *S. aureus *were reported to be Meticillin resistant based on phenotypical tests used in a study conducted in Riyadh Armed Forces Hospital among which 39 0ut of 42 carried *the mec-A* gene (12). In Iran, based on the prevalence of *mec-A* gene, the prevalence of 87.36% MRSA infection was reported in burn patients in a hospital in Ahvaz (South of Iran) (2).

However, using MIC method, about 90% of samples isolated from patients in ICU and infectious disease ward in a hospital in Tehran were Meticillin resistant (13). The prevalence of Meticillin resistant strains detected by phenotypical methods was about 39.2% in Shariati Center among which 92 isolates (93.6%) carried *mec-A* gene, that in some aspects is similar to the report from Saudi Arabia (12).

The epidemiology of MRSA has changed and it is no longer considered as a solely nosocomial pathogen (14, 15). With increasing frequency, MRSA is also detected in community and is called community-acquired MRSA (C.A MRSA) (15). A history of hospital admission has been suggested as a major risk factor for isolation of MRSA at the time of admission. In the current study the patients who had a history of admission three months before the test had shown a significantly higher rate of MRSA (P = 0.006). Furthermore, a significant difference was observed in prevalence of MRSA isolated in different wards, which indicated that the emergency department and intensive care unit had the highest rate of MRSA isolates. 

Among 46 infected patients in the emergency department, only one case had a history of previous admission. It seems that the high prevalence of MRSA infections in emergency department can be explained by the fact that because our hospital is a tertiary center where most of the patients (even with or without history of hospitalization) have prior interventions such as intravenous therapy, specialized nursing care at home or ambulatory care visits which can be easily ignored by the patient while asking about prior hospitalization. Tacconelli et al. (15) believed most C.A MRSA are not truly community acquired and this term can be confusing in the health care associated situations, and he seems to be right. 

Considering that the truly C.A MRSA strains tend to be more susceptible to a wide range of antibiotics, and genetically distinct from health care associated strains (15), it seems highly important to discriminate between these two, which can be made mostly based on the patients` history. This requires more detailed questions emphasizing on previous intravenous therapies or specialized nursing cares. Some other studies indicated organ transplantation, previous hospitalization within the past one year, and requirement for feeding tube(15) or older age (4) as risk factors of MRSA colonization.

The necessity of MRSA colonization screening at admission time is controversial. For example, Talon et al., (16) believe that systemic screening for early recognition is useful. However, some authors considered that screening of only the high risk patients would be sufficient (4) and more cost effective but requires establishment of risk factors. The current study findings including high prevalence of MRSA infections in emergency unit suggest that in a referral center like Shariati Hospital, screening the patients admitted to the emergency unit may be useful.

Considering PCR based method as the gold standard to identify the methicillin resistant *S. aureus*, the prevalence of MRSA was 36.8% in Shariati Hospital. Of course, more importantly, in order to differentiate the true C.A and Health care associated MRSA, it is recommended to ask more detailed questions about previous intra-venous therapy, history of organ transplantation, ambulatory nursing cares or antibiotic therapy, even when there is no history of prior hospital admission, since they can be easily missed if are not asked separately.
